# A Hidden Cause of Chest Pain in Adolescence: First Rib Fracture Without Trauma

**DOI:** 10.7759/cureus.85818

**Published:** 2025-06-11

**Authors:** Koji Miura, Rica Tada

**Affiliations:** 1 General Surgery, Sakaide City Hospital, Sakaide, JPN; 2 Emergency Medicine, Kagawa Prefectural Central Hospital, Takamatsu, JPN

**Keywords:** adolescent, atypical chest pain, chest pain, diagnostic imaging, first rib fracture, low-energy trauma

## Abstract

First rib fractures in adolescents are uncommon and typically result from high-impact trauma. However, they can also occur following low-energy mechanisms and may be easily overlooked in the absence of a clear traumatic event. We report the case of a 13-year-old boy with no prior medical history who presented with sudden-onset left-sided chest pain after pushing his bicycle while making a right turn. The physical examination revealed localized tenderness in the upper left chest, with no visible signs of trauma. A chest radiograph demonstrated a fracture of the left first rib. This case highlights the importance of considering first rib fractures in adolescents presenting with unexplained chest pain, even after seemingly benign activities. Prompt recognition and appropriate imaging can facilitate accurate diagnosis, avoid unnecessary testing, and alleviate anxiety for both patients and their families.

## Introduction

Fractures of the first rib in adolescents are rare and are typically associated with high-energy trauma, such as motor vehicle collisions, contact sports, or significant falls [1-3]. Due to the anatomical protection provided by the surrounding musculature and the clavicle, a considerable amount of force is generally required to produce such an injury. Nevertheless, several case reports have documented first rib fractures resulting from minor or non-traumatic activities, such as stretching or mild exertion [4-10].

In the emergency department, chest pain in adolescents is often perceived as non-urgent [11], and the absence of a clear traumatic mechanism may lead to misdiagnosis or delayed recognition of serious underlying injuries. This report describes an unusual case of a first rib fracture in a 13-year-old boy following a low-impact activity, underscoring the importance of considering this diagnosis even in the absence of trauma.

## Case presentation

**Figure 1:**
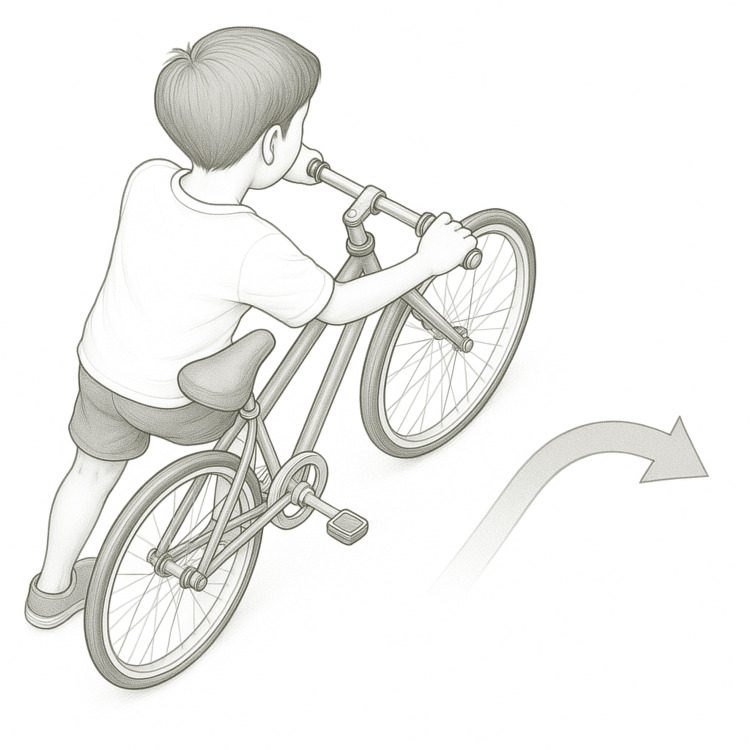
Proposed mechanism of injury. The boy is shown leaning forward while pushing the bicycle, with his left arm extended to turn the handlebar to the right. Image Credits: Koji Miura

A previously healthy 13-year-old boy presented to the emergency department with sudden-onset, left-sided chest pain accompanied by a snapping sensation. The symptoms began while he was pushing his bicycle and making a right turn (Figure 1).

He denied any history of trauma, falls, strenuous activity, or recent infections. He had no relevant past medical history and was not taking any medications.

On presentation, his vital signs were within normal limits: temperature 36.7°C, heart rate 84 beats per minute, respiratory rate 18 breaths per minute, blood pressure 112/68 mmHg, and oxygen saturation 99% on room air. The physical examination revealed localized tenderness in the upper left anterior chest wall, just beneath the medial clavicle. There were no visible signs of bruising, swelling, or deformity. Cardiac and pulmonary examinations were unremarkable.

Diagnostic assessment

**Figure 2:**
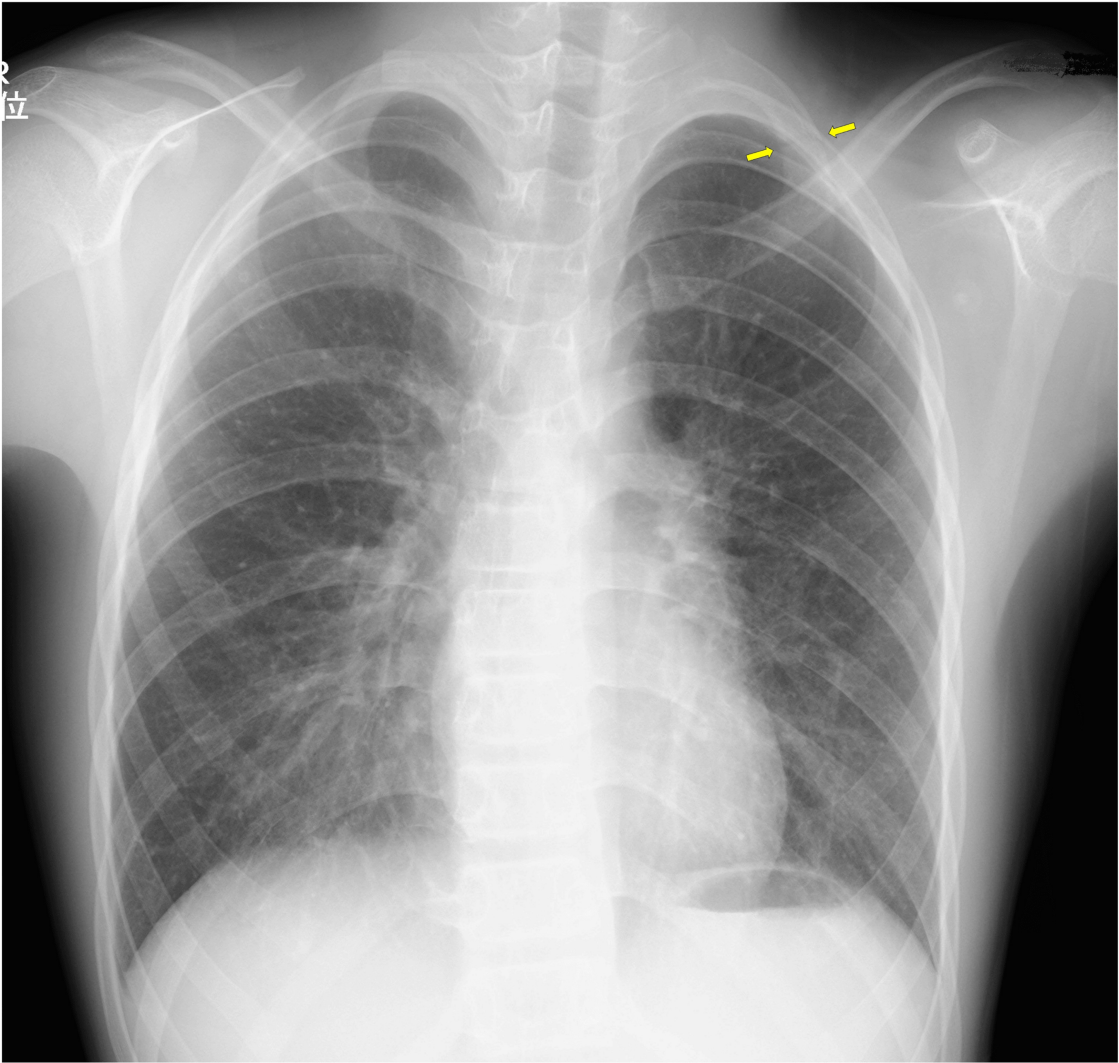
Chest radiograph. A fracture line is visible in the left first rib (yellow arrows).

In the absence of an external traumatic event, the initial differential diagnosis included spontaneous pneumothorax, musculoskeletal strain, costochondritis, and, less commonly, a stress fracture. Electrocardiography and pulse oximetry results were within normal limits. Comprehensive blood work, including serum calcium levels, was performed to evaluate for systemic causes, all of which were also within normal limits. A chest radiograph revealed a distinct fracture line through the left first rib (Figure 2), with no other osseous or soft tissue abnormalities noted.

Treatment and outcome

The patient was diagnosed with a non-traumatic first rib fracture and was managed conservatively. Acetaminophen was prescribed for pain control, and he was advised to avoid heavy lifting, contact sports, and other strenuous physical activities for several weeks. At the one-month follow-up, the patient reported significant improvement in symptoms, with no residual pain or functional limitations, indicating satisfactory healing without complications.

## Discussion

Because of their deep anatomical location and the protection provided by surrounding structures such as the clavicle, scapula, and dense musculature, first rib fractures in adolescents are typically associated with significant blunt trauma. These injuries most commonly result from high-impact accidents or substantial falls that transmit considerable force to the thoracic cage [1-3]. However, as this case illustrates, such fractures can also occur in the absence of direct trauma, following minimal exertion [4-10]. The proposed mechanism involves a sudden, forceful contraction of muscles attached to the first rib, namely, the scalene and serratus anterior, which may generate sufficient stress to cause a fracture, particularly in a developing adolescent skeleton [10]. In this instance, the patient developed symptoms while walking alongside his bicycle and making a right turn, an activity that involved a slightly forward-leaning posture with substantial extension of the left arm but without any impact or fall.

Adolescents occasionally present to the emergency department with chest pain as their primary complaint. These cases are often perceived as non-urgent, especially in the absence of trauma or abnormal vital signs, which can lead to underestimation of potentially significant injuries [11]. In rare cases, even routine physical activity can result in structural injuries such as rib fractures, which may be overlooked without a high index of suspicion. When undiagnosed, such injuries can lead to prolonged pain, anxiety, and delayed recovery.

Importantly, when a first rib fracture is suspected, a standard chest radiograph is often sufficient for diagnosis. In our case, imaging promptly revealed a clear fracture line, enabling conservative management and timely reassurance. Early recognition not only prevents unnecessary investigations, such as cardiac or pulmonary imaging, but also minimizes the risk of misdiagnosis and reduces distress for both the patient and their family.

Clinicians should maintain a high index of suspicion for first rib fractures in adolescents presenting with deep, localized chest pain beneath the medial clavicle, even in the absence of obvious trauma. Although rare, these fractures are readily identifiable with simple imaging and typically heal well with conservative treatment. Incorporating awareness of such atypical presentations into clinical practice can enhance diagnostic accuracy and improve patient outcomes.

## Conclusions

First rib fractures can occur in adolescents even in the absence of major trauma. Clinicians should consider this diagnosis when evaluating unexplained chest pain, particularly in the area beneath the medial clavicle. Early recognition using basic imaging not only facilitates appropriate management but also provides reassurance to patients and families while avoiding unnecessary escalation of care.
